# Predictors of stunting among children age 6–59 months in Ethiopia using Bayesian multi-level analysis

**DOI:** 10.1038/s41598-021-82755-7

**Published:** 2021-02-12

**Authors:** Amare Muche, Lemma Derseh Gezie, Adhanom Gebre-egzabher Baraki, Erkihun Tadesse Amsalu

**Affiliations:** 1 Department of Epidemiology and Biostatistics, School of Public Health, College of Medical and Health Sciences, Wollo University, Dessie, Ethiopia; 2 Department of Epidemiology and Biostatistics, Institute of Public Health, College of Medicine Health Sciences, University of Gondar, Gondar, Ethiopia

**Keywords:** Diseases, Health care, Medical research, Risk factors

## Abstract

In developing countries including Ethiopia stunting remained a major public health burden. It is associated with adverse health consequences, thus, investigating predictors of childhood stunting is crucial to design appropriate strategies to intervene the problem stunting. The study uses data from the Ethiopian Demographic and Health Survey (EDHS) conducted from January 18 to June 27, 2016 in Ethiopia. A total of 8117 children aged 6–59 months were included in the study with a stratified two stage cluster sampling technique. A Bayesian multilevel logistic regression was fitted using Win BUGS version 1.4.3 software to identify predictors of stunting among children age 6–59 months. Adjusted odds ratio (AOR) with 95% credible intervals was used to ascertain the strength and direction of association. In this study, increasing child’s age (AOR = 1.022; 95% CrI 1.018–1.026), being a male child (AOR = 1.16; 95%CrI 1.05–1.29), a twin (AOR = 2.55; 95% CrI 1.78–3.56), having fever (AOR = 1.23; 95%CrI 1.02–1.46), having no formal education (AOR = 1.99; 95%CrI 1.28–2.96) and primary education (AOR = 83; 95%CrI 1.19–2.73), birth interval less than 24 months (AOR = 1.40; 95% CrI 1.20–1.61), increasing maternal BMI (AOR = 0.95; 95% CrI 0.93–0.97), and poorest household wealth status (AOR = 1.78; 95% CrI 1.35–2.30) were predictors of childhood stunting at individual level. Similarly, region and type of toilet facility were predictors of childhood stunting at community level. The current study revealed that both individual and community level factors were predictors of childhood stunting in Ethiopia. Thus, more emphasize should be given by the concerned bodies to intervene the problem stunting by improving maternal education, promotion of girl education, improving the economic status of households, promotion of context-specific child feeding practices, improving maternal nutrition education and counseling, and improving sanitation and hygiene practices.

## Introduction

Stunting is a manifestation of severe, irreversible physical, physiological and cognitive impairment due to chronic malnutrition in the early life^1,2^.

Stunting causes the death of one million children each year in the world^3^. Globally, 151 million (22%) under-five children were stunted according to the recent report by UNICEF/ World Bank. Majority (91%) of stunted children were from poorest regions of the world i.e. South east Asia and Sub-Saharan Africa regions including Ethiopia^4^.

Even if the level of stunting is reduced across the world, the problem of stunting remained high in Africa. In Africa the number of stunted children is estimated to rise from 56 million in 2010 to 61 million by 2025^4^.

Further, the majority of stunted children were found in Ethiopia^5^. In the country 40% children were stunted based on report from the 2014 mini Ethiopian Demographic and Health Survey (EDHS)^6^. In addition, based on EDHS 2016 report about 38% of stunted children were found in Ethiopia^7^.

Childhood stunting is linked to different adverse health consequences and irreversible damages. It is correlated with poor developmental attainment and intelligence in children^8–10^. There is also evidence that stunted children are less likely to enroll in school and increased the risk of mortality and susceptibility to infection^4,11^. Reduced productivity, increased risk of extra weight gain and chronic non-communicable diseases in later life were reported in previous studies^10–12^. Moreover, stunting increased obstetric risk and leads to low birth weight babies thus, have an intergenerational impact^10,13^.

According to studies done previously in different parts of Ethiopia large family size, multiple siblings, food insecurity, poor wealth status, inadequate health care utilization and sanitary practices, unavailability of latrine and unprotected source of water were significant predictors of childhood stunting^14–22^. Stunting is also reported with children whose mothers are illiterate^23^. Male sex, frequent diarrheal episode and doesn’t receive vitamin A supplementation also increases the likelihood of stunting^24–27^.

Even though the government of Ethiopia adopted and implemented the national nutrition strategy, national nutrition program, and infant and young child feeding intervention programs to reduce the problem in the past few years, still stunting remained a major public health burden^6,7,28–30^.

However, previous studies done on stunting in different parts of Ethiopia more focused on individual level factors associated with stunting by employing basic regression models. In this study community level factors were included and addressed by using advanced mixed effect model which is appropriate for hierarchical DHS data. In addition, most of these studies were conducted in small localized areas and using small sample size which were not representative figure at the national level due to variations in socioeconomic, culture, awareness and practices in different parts of Ethiopia. But the current study used the national representative data in all regions of the country from Ethiopia Demographic Health Survey 2016 (EDHS) data to provide the national figure.

Furthermore, previous studies regarding predictors of stunting were used the classical/frequents statistical methods. In this study the Bayesian approach was used to estimate parameters of interest. Thus, employing Bayesian statistical method for parameters estimation is superior when multilevel logistic regression model is employed compared to the classical methods. Therefore, this study aims to investigate predictors of stunting among children aged 6–59 months in Ethiopia using multi-level analysis by applying Bayesian statistical approach which has powerful estimation for hierarchical data and generates evidence from prior information and the current data.

## Results

### Socio-demographic characteristics of respondents

A total of 8117 children age 6–59 months were included in the analysis. The median age of children were 31 months (IQR = 18–46). The mean maternal BMI was 20.70 kg/m^2^ with (SD ± 3.40). In addition, the mean height of mothers was 158 cm with (SD ± 6.7). Most of the study participants about, 7242 (89.22%) were rural residents.

Regarding family wealth index about 1901(23.42%) of respondents were from the poorest house.

Majority of children about, 7020 (86.49%) belongs to male household head. Regarding maternal education, nearly two third, 5388 (66.38%) of children had mothers who have no formal education.

In this study about, 3427 (42.96) of respondents were not anemic. Of the children, 7109 (87.59%) had no history of diarrhea and about, 4429 (54.57%) were not received vitamin A supplementation in the last 6 months.

Most of the households about, 7353 (90.59%) had not used improved toilet facilities, and about, 4582 (56.45%) of the households obtained water from improved sources (Table 1).

**Table 1:** Socio-demographic and other characteristics of children aged 6–59 months in Ethiopia from January 18, 2016 to June 27, 2016 (n = 8117).

Variables	Weighted frequency	Weighted percent
**Sex of child**		
Female	3933	48.46
Male	4184	51.54
**Mother’s education level**		
No education	5388	66.38
Primary education	2216	27.30
Secondary education	340	4.19
Higher	173	2.14
**Wealth index**		
Richest	1130	13.93
Richer	1456	17.94
Middle	1742	21.46
Poorer	1888	23.26
Poorest	1901	23.42
**Head of household**		
Female	1097	13.51
Male	7020	86.49
**Residence**		
Urban	875	10.78
Rural	7242	89.22
**Drinking water source**		
Improved	4582	56.45
Unimproved	3535	43.55
**Time to get drinking water**		
< 30 min	3849	47.42
≥ 30 min	4268	52.58
**Type of toilet facility**		
Improved	764	9.41
Unimproved	7353	90.59
**Birth interval**		
< 24 months	1720	21.18
≥ 24 months	6397	78.82
**Type of birth**		
Single	7922	97.59
Multiple	195	2.41
**Diarrhea in the last 2 weeks**		
Yes	1008	12.41
No	7109	87.59
**ARI in the last 2 weeks**		
Yes	1629	20.07
No	6488	79.93
**Fever in the last 2 weeks**		
Yes	1209	14.90
No	6908	85.10
**Anemia level (child)**		
Not anemic	3427	42.96
Mild	247	3.10
Moderate	2305	28.90
Severe	1998	25.05
**Vitamin A supplementation in the last 6 months**		
Yes	3688	45.43
No	4429	54.57
**Region**		
Addis Ababa	181	2.23
Dire Dawa	32	0.39
Tigray	567	6.91
Afar	80	0.98
Amhara	1561	19.30
Oromia	3498	43.10
Somali	340	4.19
Benishangul-Gumuz	86	1.06
SNNPR	1736	21.39
Gambela	19	0.24
Harari	17	0.21
**Household family size**		
≤ 5	3544	43.66
> 5	4573	56.34
**No of under-five children in the HH**		
1	3006	37.04
2	3755	46.26
3	1147	14.13
≥ 4	209	2.57

In this study the overall prevalence of stunting among children aged 6–59 months in Ethiopia was 41.20% (95% CI 40.16%, 42.25%), comprising of moderate stunting 22.41% (95% CI 21.54%, 23.31%) and severe stunting 18.79% (95% CI 18.00%, 19.64%).

Childhood stunting was more prevalent in males 1838 (44%) than females 1505 (38%). Also, the likelihood of stunting was highest in children from mothers who had no formal education 2388 (41%). Similarly the magnitude of stunting was higher among children from the poorest households 925 (49%) and who had mild anemia 130 (52.5%) (Table 2).

**Table 2:** Distribution of stunting by the selected characteristics among children aged 6–59 months in Ethiopia from January 18, 2016 to June 27, 2016 (n = 8117).

Variables	Stunted (HAZ < − 2 SD)	Normal (≥ − 2 SD)	Total
**Sex of child**			
Female	1505 (38.26%)	2428 (61.74%)	3933(48.46%)
Male	1838 (43.93%)	2346 (56.07%)	4184(51.54%)
**Type of birth**			
Single	3226 (40.75%)	4696 (59.27%)	7922 (97.59%)
Multiple	116 (59.46%)	79 (40.54%)	195 (2.41%)
**Mother’s education level**			
No education	2388 (41.18%)	3000 (55.68%)	5388 (66.38%)
Primary education	843 (38.01%)	1373 (61.99%)	2216 (27.30%)
Secondary education	85 (25.06%)	255 (74.94%)	340 (4.19%)
Higher	27 (15.64%)	146 (84.36%)	173 (2.14%)
**Wealth quintile**			
Poorest	925 (48.66%)	976 (51.34%)	1901 (23.42%)
Poorer	872 (46.17%)	1016 (53.83%)	1888 (23.26%)
Middle	701 (40.26%)	1041 (59.74%)	1742 (21.46%)
Richer	549 (37.71%)	907 (62.29%)	1456 (17.994%)
Richest	295 (26.15%)	835 (73.85%)	1130 (13.93%)
**Anemia level (child)**			
Not anemic	1283 (37.44%)	2144 (62.56%)	3427 (42.96%)
Mild	130 (52.50%)	117 (47.50%)	247 (3.10%)
Moderate	1018 (44.15%)	1287 (55.85%)	2305 (28.90%)
Severe	866 (43.35%)	1132 (56.65%)	1998 (25.05%)
**Type of toilet facility**			
Improved	214 (28.07%)	550 (71.93%)	764 (9.41%)
Unimproved	3128 (57.46%)	4225 (57.46%)	7353 (90.59%)
**Region**			
Addis Ababa	29 (15.96%)	152 (84.04%)	181 (2.23%)
Dire Dawa	14 (43.40%)	18 (56.60%)	32 (0.39%)
Tigray	233 (41.47%)	328 (58.53%)	567 (6.91%)
Afar	35 (44.05%)	45 (55.95%)	80 (0.98%)
Amhara	805 (51.38%)	762 (48.62%)	1561 (19.30%)
Oromia	1351 (38.63%)	2147 (61.37%)	3498 (43.10%)
Somali	100 (29.31%)	241 (70.69%)	340 (4.19%)
Benishangul-Gumuz	39 (45.40%)	47 (54.60%)	86 (1.06%)
SNNPR	726 (41.84%)	1010 (58.16%)	1736 (21.39%)
Gambela	5 (25.31%)	14 (74.69%)	19 (0.24%)
Harari	6 (34.92%)	11 (65.08)	17 (0.21%)

### Predictors of stunting among children age 6–59 months in Ethiopia

In the Bayesian multi-level multivariate logistic regression analyses model both individual and community level factors were included.

Among individual level factors sex of child, child age, type of birth, anaemia status of the child, history of fever in the last 2 weeks, education level of the mother, maternal BMI, maternal height, birth interval, and family wealth index were identified as significant predictors of childhood stunting. Among community level factors included in the study only type of toilet facility and region were significant predictors of childhood stunting.

### Individual level factors

The Bayesian multi-level multivariable logistic regression analysis result showed that being male children were 16% more (AOR = 1.16, 95% CrI 1.05–1.29) likely to be stunted as compared to female children.

Children of multiple birth (twin) was 2.55 times (AOR = 2.55, 95% CrI 1.78–3.56) more likely to be stunted as compared to their counterpart. Children who had fever were 23% (AOR = 1.23, 95% CrI 1.02–1.46) more likely to be stunted compared to the counterparts.

Children with mild anemia were three times (AOR = 3.13, 95% CrI 2.34–4.12), moderate anemia 81% (AOR = 1.81, 95% CrI 1.58–2.08), and severe anemia 37% (AOR = 1.37, 95% CrI 1.19–1.56) more likely to be stunted compared to children who were not anemic.

Children belonging to mothers who had no formal education were 99% (AOR = 1.99, 95% CrI 1.28–2.96), and primary education were 83% (AOR = 1.83, 95% CrI 1.19–2.73) more likely to be stunted compared to children whose mothers had higher education.

Children from poorest house hold wealth status were 78% (AOR = 1.78, 95% CrI 1.35–2.30), poorer household 73% (AOR = 1.73, 95% CrI 1.31–2.23), and middle household 37% (AOR = 1.37, 95% CrI 1.04–1.78) more likely to be stunted compared to children from richest household wealth status.

The odds of being stunted were 40% (AOR = 1.40; 95% CrI 1.20–1.61) higher among children with < 24 months birth interval compared to children having ≥ 24 months birth interval (Table 3).

**Table 3:** Multi-level logistic regression analysis of factors associated with childhood stunting in Ethiopia by using Bayesian statistical inference from January 18, 2016 to June 27, 2016 (n = 8117).

Variables	Model 2 AOR (95% Crl*)	Model 3 AOR (95% Crl*)	Model 4 AOR (95% Crl*)
Child age (months)	1.022 (1.018–1.025)*		1.022 (1.018–1.026)*
**Sex of child**			
Female	1.00		1.00
Male	1.16 (1.04–1.28)*		1.15 (1.02–1.28)
**Type of birth**			
Single	1.00		1.00
Multiple (twin)	2.61 (1.82–3.63)*		2.77 (1.87–3.97)
**Diarrhea**			
No	1.00		1.00
Yes	1.21 (1.02–1.42)*		1.19 (0.99–1.40)
**ARI**			
No	1.00		1.00
Yes	0.94 (0.79–1.01)		0.93 (0.78–1.09)
**Fever**			
No	1.00		1.00
Yes	1.22 (1.01–1.45)*		1.23 (1.02–1.46)
**Anemia level (child)**			
Not anemic	1.00		1.00
Mild	1.65 (1.26–2.15)*		3.13 (2.34–4.12)
Moderate	1.25 (1.19–1.58)*		1.81 (1.58–2.08)
Severe	1.16 (1.12–1.38)*		1.37 (1.19–1.56)
**Vitamin A**			
Yes	1.00		1.00
No	0.96 (0.86–1.06)		1.00 (0.89–1.12)
**Mother’s education**			
Higher education	1.00		1.00
Secondary	1.73 (1.08–2.76)*		1.57 (0.98–2.41)
Primary	2.11 (1.37–3.30)*		1.83 (1.19–2.73)
No education	2.30 (1.49–3.59)*		1.99 (1.28–2.96)
Mother’s BMI (kg/m^2^)	0.95 (0.93–0.97)*		0.95 (0.93–0.97)
Maternal height (cm)	0.94 (0.93–0.96)*		0.945 (0.936–0.954)
**Wealth quintiles**			
Richest	1.00		1.00
Richer	1.33 (1.06–1.64)*		1.19 (0.90–1.54)
Middle	1.54 (1.23–1.90)*		1.37 (1.04–1.78)
Poorer	1.92 (1.54–2.35)*		1.73 (1.31–2.23)
Poorest	1.85 (4.85–10.48)*		1.78 (1.35–2.30)
**Head of household**			
Male	1.00		1.00
Female	1.01 (0.88–1.15)		1.06 (0.92–1.21)
**Birth interval**			
≥ 24 months	1.00		1.00
< 24 months	1.35 (1.20–1.56)*		1.40 (1.20–1.61)
**Household family size**			
≤ 5	1.00		1.00
> 5	1.06 (0.94–1.18)		1.07 (0.95–1.19)
**No of under 5 children**			
1	1.00		1.00
2	1.05 (0.93–1.18)		0.94 (0.83–1.05)
3	0.73(0.61–0.86)*		0.73 (0.62–1.03)
≥ 4	0.72 (0.50–1.01)		0.75 (0.52–1.04)
**Drinking water source**			
Improved		1.00	1.00
Unimproved		0.99 (0.88–1.11)	0.91 (0.80–1.03)
**Time to get drinking water**			
< 30 min		1.00	1.00
≥ 30 min		1.06 (0.94–1.20)	1.08 (0.94–1.23)
**Residence**			
Urban		1.00	1.00
Rural		1.55 (1.26–1.89)	0.87 (0.66–1.13)
**Type of toilet facility**			
Improved		1.00	1.00
Unimproved		1.61 (1.34–1.92)	1.26 (1.05–1.54)
**Region**			
Addis Ababa		1.00	1.00
Dire Dawa		2.62 (1.64–3.99)*	2.34 (1.41–3.67)
Tigray		2.26 (1.45–2.21)*	2.05 (1.28–3.13)
Afar		2.76 (1.75–4.12)*	1.85 (1.13–2.87)
Amhara		2.97 (1.90–4.41)*	2.81 (1.73–4.33)
Oromia		1.65 (1.05–2.43)*	1.86 (1.03–1.78)
Somali		1.30 (0.84–1.91)	1.23 (0.76–1.88)
Benishangul-Gumuz		2.38 (1.49–3.58)*	2.46 (1.50–3.83)
SNNPR		1.95 (1.25–2.88)*	2.01 (1.25–3.06)
Gambela		1.18 (0.74–1.79)	1.36 (0.82–2.13)
Harari		1.72 (1.06–2.63)*	1.93 (1.02–3.42)
σ^2^_u_	0.25 (0.17–0.34)	0.23 (0.16–0.31)	0.20 (0.13–0.29)
σ^2^_e_	4.15 (2.99–5.83)	4.46 (3.21–6.21)	5.11 (3.50–7.60)
VPC^a^	0.07 (0.05–0.09)	0.07 (0,05–0.09)	0.06 (0.04–0.08)
Deviance^b^	8821 (8753–8893)	9503 (9436–9572)	8806 (8738–8876)
DIC^d^	9061.900	9732.760	9030.030

Crl*: is Bayesian Credible Interval, σ^2^_u_: is residual variance between clusters_,_ σ^2^_e_: residual variance between individuals in the same cluster_,_ VPC^a^: is variance partition coefficient, Deviance^b^: is − 2log (L), DIC^d^: is deviance information criterion (model fit statistic).

### Community level factors

The finding of this study indicated that type of toilet facility (i.e. unimproved) and region were significant predictors of childhood stunting at community level.

Those children from households who had unimproved toilet facility was 26% (AOR = 1.26; 95% CrI 1.05–1.54) more likely to be stunted compared to children from household who had access to improved toilet facility.

Similarly region was also found to be significant predictors of childhood stunting. Hence the likelihood of stunting among children living in Dire-Dawa were 2.34 (AOR = 2.34; 95% CrI 1.41–3.67), Afar 1.85 (AOR = 1.85; 95% CrI 1.13–2.87), Amhara 2.81 (AOR = 2.81; 95% CrI 1.73–4.33), Benishangul-Gumuz 2.46 (AOR = 2.46; 95% CrI 1.50–3.83), Harari1.93 (AOR = 1.93; 95% CrI 1.01–3.42), Oromia 1.86 (AOR = 1.86; 95% CrI 1.03–1.78), SNNPR 2 (AOR = 2.01; 95% CrI 1.25–3.06), and Tigray 2.1 (AOR = 2.05; 95% CrI 1.28–3.13) times higher compared to children live in Addis Ababa (Table 3).

## Methods

### Study area and period

The 2016 Ethiopian Demographic and Health Survey (EDHS) is the fourth Demographic and Health Survey conducted in Ethiopia, which was conducted from January 18, 2016 to June 27, 2016. The sampling frame used for the 2016 EDHS is the Ethiopia Population and Housing Census (PHC), which was conducted in 2007 by the Ethiopian Central Statistical Agency. The census frame is a complete list of 84,915 enumeration areas (EAs) created for the 2007 PHC. An EA is a geographical area covering on average 181 households^7^.

### Study design

A Community-based cross-sectional study design was conducted among children aged 6–59 months.

### Population and sample

All children aged 6–59 months resided in Ethiopia (nine regions and two city administrations) were the source population and the study population was all children aged 6–59 months who were living in the selected households of Ethiopia. A total of 8117 children age 6–59 months which fulfil the inclusion criteria were included in the analysis.

**Figure 1:**
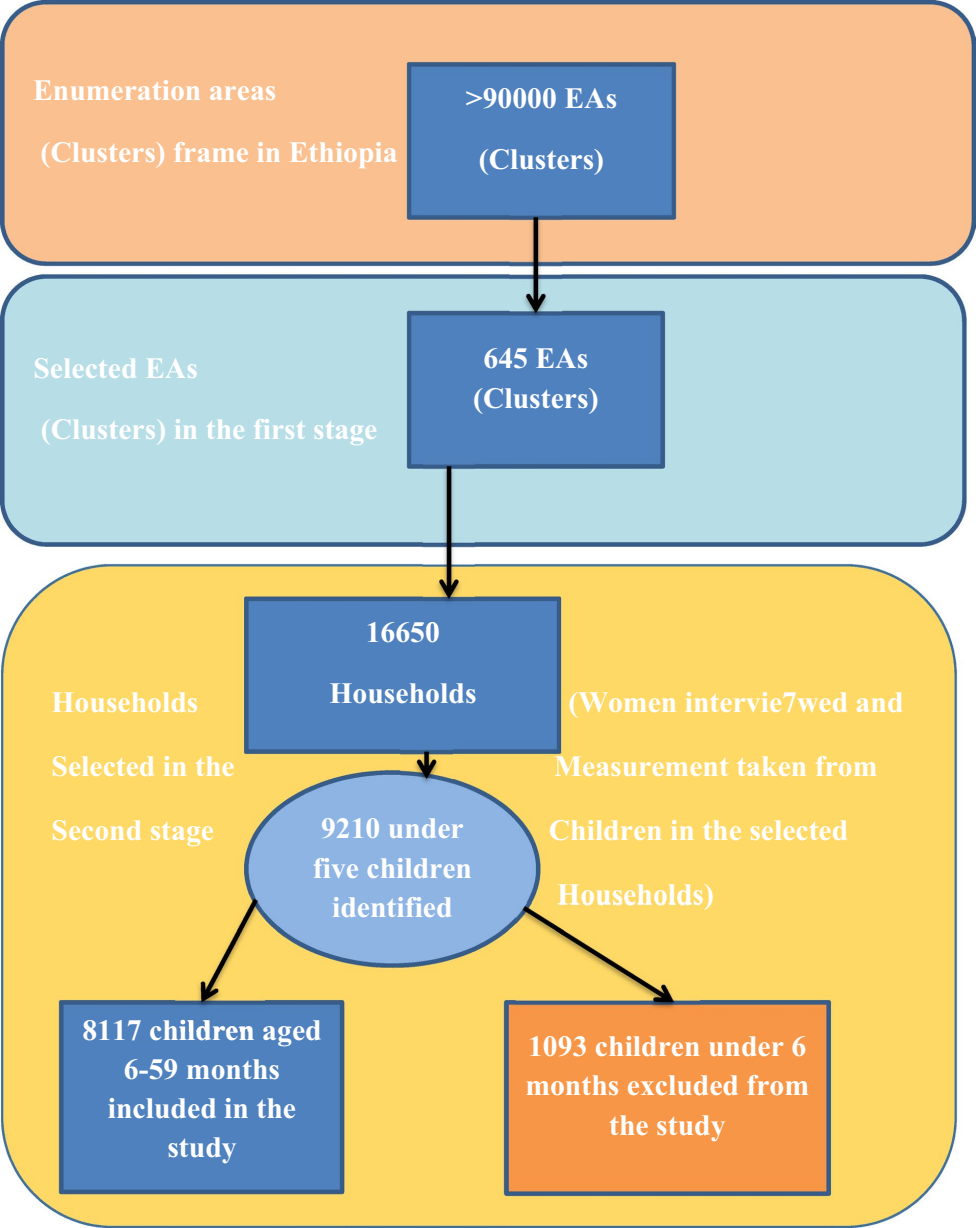
The sampling procedure of the study predictors of stunting among children aged 6–59 months in Ethiopia (n = 8117).

A stratified two-stage cluster sampling procedure was employed where enumeration areas (EA) were the sampling units for the first stage and households for the second stage. In the 2016 EDHS, a total of 645 EAs (202 urban and 443 rural) were selected with a probability proportional to EA size (based on the 2007 housing and population census) and independent selection in each sampling stratum. Of these, 18,008 households and 16,583 eligible women were included. The detailed sampling procedure was presented in the full EDHS report^7^ indicated in the Fig. 1 below.

### Data collection procedure and variables

Stunting among children 6–59 months age was the dependent variable of the study. The independent variables were community level factors (region, clusters, residence, source of drinking water, and type of toilet facility), household factors (wealth index, household head family size, and number of under five children in the house hold), maternal factors(educational level, marital status, body mass index (BMI), maternal height, ANC visit, and birth interval), and child factors (age, sex, birth type, birth order, birth weight, diarrhea, acute respiratory tract infection (ARI), fever, vitamin A supplementation, and anemia status).

Stunting is defined as children who have low height/length-for-age-Z score < − 2 SDs of the median value of the WHO Child Growth Standards median aged 6–59 months^1,31^.

Improved drinking water source includes piped water, public tap, standpipes, tube wells, boreholes, protected dug wells and spring, rain water, and bottled water^32^. Improved toilet facility includes any non-shared toilet of those types: flush/pour flush toilets to piped water systems, septic tanks and, and pit latrines; ventilated improved pit (VIP) latrines, pit latrines with slabs, and composting toilets^32^. Unimproved toilet facility includes flush to somewhere else, flush do not know where, pit latrine without slab/open pit, no facility/bush/field, bucket toilet, hanging toilet/latrine, others^32^.

Anemia level was determined based on hemoglobin levels adjusted for altitude in enumeration areas that are above 1000 m. They are classified as not anemic if the hemoglobin level is ≥ 11.0 mg/dl, mild anemic if hemoglobin level is from 10.0–10.9 mg/dl, moderate anemic if the level of hemoglobin is from 7.0–9.9 mg/dl, and severe anemic if the level of hemoglobin is < 7.0 mg/dl)^7^.

Body mass index (BMI) for women aged 15–49 years who are not pregnant and who have not had a birth in the last 2 months before the survey was determined by divided weight in kilograms by height in meters square (kg/m^2^). Accordingly classified as under-weight/thin (BMI < 18.5 kg/m^2^), normal (BMI in the range of 18.5–24.9 kg/m^2^) and over-weight (BMI ≥ 25.0 kg/m^2^)^7^.

### Anthropometric measurements

The length of children aged < 24 months was measured during the EDHS in a recumbent position to the nearest 0.1 cm using a locally made measuring board (Shorr Board) with an upright wooden base and moveable headpieces. Children ≥ 24 months were measured while standing upright. The length/height-for-age Z-score, an indicator of nutritional status, was compared with reference data from the WHO Multicenter Growth Reference Study Group, 2006^33^. Children whose height-for-age Z-score is < − 2 SD from the median of the WHO reference population are considered stunted (short for their age).

Weight measurement was taken after children were undressed (no shoe, dresses and wet hat). For a child who stands on the weighing scale calmly, the measurement was taken in the nearest 0.1 kg. In the time of refuse to be scaled, children’s mother carried and stood on the scale. Finally, the child actual weight was registered by subtracting mother’s weight from mother and child weight^7^.

Before the actual data collection, interviewers were trained and a pre-test was performed. Interviews were also performed using local languages. A structured and pre-tested questionnaire was used as a tool for data collection. The 2016 EDHS interviewers used tablet computers to record responses during interviews. The tablets were equipped with Bluetooth technology to enable remote electronic transfer of files (transfer assignment sheets from team supervisors to interviewers and transfer of completed copies from interviewers to supervisors)^7^.

### Data management and analysis

The data set was available at the DHS website https://www.dhsprogram.com/data/available-datasets.cfm. After registering for the permission it was accessed through the DHS website; https://dhsprogram.com/data/dataset_admin/login_main.cfm. The kid recode (KR) data set in STATA file contains the outcome and predictor variables of the study. The data was explored, cleaned, coded, re-categorized and recoded. Then, the data was prepared in text file for analysis using publicly available software Win BUGS. Finally, in the Win BUGS program the distributional form of the data and the parameters were specified. Model specification in terms of the distributional relationships between observables and parameters (likelihood) and prior distributions of parameter, auxiliary files containing the data and initial values for unknowns were input to Win BUGS.

This study was based on secondary data analysis of 2016 EDHS by adjusting sample weights. Categorical characteristics and outcome of the study was described in terms of percentage and frequencies. Tables were used to present the result. A Bi-variable multi-level logistic regression analysis was carried out to see the crude effect of each independent variable on stunting, and then variables with P < 0.2 were entered to the multivariable multi-level logistic regression analysis^34^.

The Bayesian multi-level logistic regression model was employed using Win BUGS statistical software package version 1.4.3 (MRC Biostatistics Unit, Cambridge and Imperial College London, UK) to identify predictors of stunting. WinBUGS is a windows-based computer program designed to conduct Bayesian Analyses of complex statistical models using Markov Chain Monte Carlo (MCMC) methods. The parameter estimation was done with MCMC simulation method by using Gibbs sampling technique. Non informative priors for fixed and random effect parameters were selected and three different initial values for these parameters were specified. Model specification with binary multi-level logistic regression was checked, data were loaded that were presented with rectangular text file format. Initial values compiled with the model and initialized.

The deviance information criterion (DIC) statistic^35,36^ was calculated for the different models (individual level, community level and both individual and community level) fitted with logit, probit and cloglog link functions. The DIC was used to evaluate and compare model performance of the full model and the reduced model. A model with lower DIC was considered as one with a better fit.

Convergence of parameters was checked visually using history plots, kernel density, auto correlation plots and Gulman-Rubin convergence diagnostic. Summary statistic (mean, SD, AOR and 95% credible intervals for parameters) from the posterior distribution of the parameters was calculated from simulated samples.

Variance partition coefficient (VPC) statistic was calculated to measure the variation between clusters (the random effect variable)^37^. It represents the percentage variance explained by higher level clusters. Hence, it was calculated as below:

$$ vpc = \frac{{\sigma_{u}^{2} }}{{(\sigma_{e}^{2 } + \sigma_{u}^{2} )}} $$

where $\sigma_{u}^{2}$ is the between cluster variance, $\sigma_{e}^{2}$ = 3.2.

### Ethical issues

Ethical clearance was gained from the ethical review board of Institute of Public Health, College of Medicine and Health Sciences, University of Gondar. Written consent was obtained from Measure DHS International Program which approved the data-sets. All the data used in this study are publicly available, aggregated secondary data with not having any personal identifying information that can be linked to particular individuals, communities, or study participants. Confidentiality of data was maintained anonymously.

### Confirmation of methods

Author(s) confirm that all methods were carried out in accordance with relevant guidelines and regulations in the manuscript.

## Discussion

The study demonstrated that child’s age was significant predictors of childhood stunting. That is the older the child the higher the risk of being stunted. This finding is in line with studies done in Ethiopia and other developing countries^38–42^. This could be due to the fact that stunting is a chronic malnutrition and is commonly noticed long term nutritional deprivation. The older children are being weaned off breast feeding and also they are becoming mobile and get contaminated materials such as water, food, and soil then take into their mouth. These conditions expose children to infection which decreases food intake^43,44^.

Our study showed that male children were more stunted compared to female children. It is similar with reports from different regions^38,40,41,45,46^. The justification may be related that in early life childhood morbidity is higher among males than females. In addition, it is related with the higher number of male preterm births as compared to female preterm births that lead to increase stunting in under-five children^47^. In contrast to this finding studies demonstrated female children were more stunted compared to male children^48^. But this discrepancy could not be important to design interventions, because sex is not modifiable factor.

Educational level of the mother showed a significant negative association with stunting among under-five children. This finding is similar with previous reports that maternal education has a positive outcome in reducing the child stunting^40,41,45,48,49^. One possible explanation is that knowledge that mothers get from their formal education could capable them to practice nutritional and other related behaviors that prevent chronic malnutrition/stunting. In addition to this, educated mothers have better health seeking behavior for childhood illnesses as compared to uneducated mothers^50^. A higher maternal education leads to better health care practice, acceptance of modern health practices and higher female autonomy, that affects health-related decisions again increases nutritional effects^51^. Further, education is one of an important tool for improvement of income which helps them to satisfy nutritional requirements. Furthermore, education increases skills and is highly linked with different socio-economic factors including life style, income, and fertility at individual and community level^8^.

In this study, maternal nutritional status was significant predictors of childhood stunting. Studies done in Ethiopia and Nigeria reported similar findings^39,40,44,52^. Maternal BMI is an essential predictor of childhood stunting and affected by maternal nutrition, hence proper nutrition for mothers during prenatal and postnatal period is highly demanded to enhance the growth of children. Maternal under-nutrition which leads poor growth of fetus leading to intrauterine growth retardation, this in turn strongly related with childhood stunting^39,53^.

Likewise, maternal height was found significant predictor of childhood stunting. The finding was consistent with studies done in India and Cambodia^54,56^. This could be explained by short maternal stature is associated with intrauterine growth retardation and low birth weight which are in turn predictors of impaired child growth^57^.

In addition, having birth interval < 24 months increases the likelihood of stunting. The finding is consistent with the previous studies^39,40,55^. The possible explanation could be those who had a short birth interval between births could be followed by adverse consequence on child nutrition because of it undermines intrauterine growth and caring quality of the child^58^.

The economic status of the households is related with nutritious foods at the household level which could determine the growth and the development of the children in early life^59^. The result of this study showed that children with households in the poorest wealth quintiles were more likely to be stunted than children with richest wealth index, which is consistent with the previous studies^40,42,45^. It is clear that, increased income improves dietary diversity, which in turn improves nutrient intake and nutritional status of the children and the mother, and it will result appropriate growth and development^60,61^.

From this study finding, children being twin were positively associated with stunting. This finding is in line with studies done in Cambodia^55^. However, the result is contradicting to studies done in India which showed being twin and stunting had negative association^62,63^. This might be due to socio-cultural differences in child care practices of the countries.

Children having fever were more likely to being stunted as compared to those children who had no fever. This may be due to the fact that during illness children have reduced intake of food and in turn affects their body nutrients requirement. Also, this study finding was opposite to studies done in Kenya, Nepal and Colombia that showed negative association between childhood stunting and fever^64–66^.

This study also showed that the likelihood of stunting was higher among children from households who had unimproved toilet facility compared to children from households who had improved toilet facility. This finding is consistent with findings from studies in Sub-Saharan Africa and Cambodia^39,55^. It is explained that children become more affected by environmental contamination when they start crawling, walking, exploring and taking objects to their mouth, that increases the risk of infection. This leads to diarrheal illness which in turn deteriorates nutritional status of children^67^. In this study region was found to be significant predictors of childhood stunting. Accordingly, children living in Dire-dawa, Amhara, Afar, Tigray, SNNPR, Oromia, and Benishangul Gumuz regions were more likely to be stunted compared to children living in Addis-Ababa. This finding is supported by studies conducted in Ethiopia^40^. In Ethiopia there are socio-cultural and socio-economic differences among regions, which influence the household food security, which in turn affect the risk of childhood stunting. In addition, the amount and distribution of rainfall and temperature influences household food availability, thus increasing the risk of a child to be stunted^68^.

Using Bayesian statistical analysis, which has high power and computes the posterior distribution which precludes the prior information with the current data and inference is based on the posterior distribution. Moreover, the use of multilevel logistic regression analysis, which was able to identify other factors beyond individual level factor that would not be identified by using standard logistic regression analysis, and a nationally representative population based study that could be made inference for all children in Ethiopia were the strength of study.

As a limitation the study didn’t include important variables such as quantitative dietary consumption behavioral factors to substantiate the findings. In addition the study did not address the causal effects, since it was based on data from cross-sectional.

The findings of the study contributes knowledge to the new statistical approach (Bayesian inference) for researchers and guide public health planners and policy makers and other concerned bodies to design appropriate intervention programs including improving maternal education, promotion of girl education, improving the economic status of households, promotion of context-specific child feeding practices, improving maternal nutrition education and counseling, improving sanitation and hygiene practices to intervene the problem of stunting. In general, these findings are of supreme importance for the Ministry of Health, health bureaus, and partners to develop nutritional programs to reduce childhood stunting.

## Conclusion

In this study, both individual and community level factors were significant predictors of childhood stunting. Accordingly, increased child’s age, being a male child, a twin, children with anemia, having fever, have no formal education and primary education of the mother, short birth interval (< 24 months) and lived in middle or lowest household wealth status were predictors increased the likelihood of childhood stunting at the individual level. On the other hand, increased maternal BMI and maternal long stature were factors that reduced the odds of childhood stunting at the individual level. Using unimproved toilet facility and living in Dire-dawa, Amhara, Afar, Tigray, SNNPR, Oromia, and Benishangul Gumuz regions increases the likelihood of childhood stunting at community level.

## Data Availability

Data is available online from http://www.measuredhs.com. A letter of approval for the use of the data was obtained from the Measure DHS and the data set was downloaded from the website (https://dhsprogram.com/data/available-datasets.cfm). This study used EDHS 2016 child data set and extracted the outcome and explanatory variables.
